# Excessive intraoperative blood loss independently predicts recurrence of hepatocellular carcinoma after liver transplantation

**DOI:** 10.1186/s12876-015-0364-5

**Published:** 2015-10-15

**Authors:** Bing Liu, Fei Teng, Hong Fu, Wen-Yuan Guo, Xiao-Min Shi, Zhi-Jia Ni, Xiao-Gang Gao, Jun Ma, Zhi-Ren Fu, Guo-Shan Ding

**Affiliations:** Department of Liver Surgery and Organ Transplantation Institute of Changzheng Hospital, Second Military Medical University, 415 Fengyang Road, Shanghai, 200003 China

**Keywords:** Hepatocellular carcinoma, Liver transplantation, Intraoperative blood loss, Recurrence

## Abstract

**Background:**

Several studies have investigated the effect of intraoperative blood loss (IBL) on recurrence of tumors. However, the independent effect of IBL on oncological outcome after liver transplantation (LT) for hepatocellular carcinoma (HCC) is unclear.

**Methods:**

A total of 479 patients who underwent LT for HCC from January 2001 to December 2012 at our institution were enrolled in this retrospective study. Kaplan–Meier and Cox regression methods were used to assess the recurrence rate, as well as its risk factors. Stratified analysis was performed to further examine the effect of IBL on HCC recurrence according to different characteristics of tumors. We also investigated the independent risk factors for excessive IBL using logistic regression analysis.

**Results:**

The median follow-up was 28 months (range, 1–131 months). Kaplan–Meier analysis with the log-rank test according to IBL at per liter intervals showed that IBL > 4 L was significantly associated with a higher recurrence rate (*P* < 0.001). Multivariate analysis identified that IBL > 4 L (*P* < 0.001; hazard ratio [HR] = 2.32, 95 % confidence interval [CI] = 1.60–3.36) was an independent risk factor for post-LT HCC recurrence, as well as age < 60 years, exceeding Milan criteria, α-fetoprotein levels > 400 ng/mL, and micro- and macrovascular invasion. IBL > 4 L (*P* < 0.001; HR = 2.45, 95 % CI = 1.64–3.66) was also independently associated with early (within 1 year) recurrence after LT. Furthermore, a significant correlation between IBL > 4 L and vascular invasion (*P* = 0.019) was found. IBL > 4 L was independently associated with HCC recurrence for patients with vascular invasion, but not for patients without vascular invasion. Finally, we found that the presence of ascites, model for end-stage liver disease score, and operation time were independent risk factors for IBL > 4 L.

**Conclusions:**

Excessive IBL is an independent predictor of HCC recurrence after LT, especially in patients with vascular invasion.

## Background

Hepatocellular carcinoma (HCC) is the third most common cause of cancer-related death and is currently the major event leading to death in patients with cirrhosis [1]. Liver transplantation (LT) is an ideal treatment for HCC because it can simultaneously cure the tumor and underlying cirrhosis. Additionally, the effectiveness of LT is not affected by the degree of impairment of liver function [2]. However, this major surgery is often accompanied by excessive intraoperative blood loss (IBL). IBL is a significant prognostic determinant for hepatocellular carcinoma [3]. as well as many other cancer types, such as gastric cancer [4], colorectal cancer [5], prostate cancer [6], and pancreas cancer [7]. Previous studies have evaluated the relationship of IBL in liver resection with outcomes in HCC patients. These studies demonstrated that IBL can independently predict survival and recurrence [3]. However, there is little research on the effect of IBL on prognosis after LT for HCC, especially the effect on HCC recurrence. Furthermore, to the best of our knowledge, no studies have further investigated the relationship between IBL and post-transplant HCC recurrence according to different tumor characteristics, such as vascular invasion status. Therefore, this study aimed to assess the relationship between IBL and HCC recurrence after LT through comprehensive evaluation.

## Methods

### Patients

Medical records of 570 patients who underwent deceased donor LT at our transplant center between January 2001 and December 2012 were reviewed retrospectively. Of these patients, 91 were excluded for various reasons as follows. Forty (7.0 %) patients were pathologically confirmed as having cholangiocellular carcinoma or mixed liver cancer, 24 (4.2 %) died within 1 month after LT without evidence of HCC recurrence, and 27 (4.7 %) had insufficient data. Finally, 479 patients were enrolled in our study. This study was approved by the Research Ethics Committee of Changzheng Hospital, Shanghai and written informed consents were obtained from all the participants. Demographic, clinical, and laboratory data were collected from the records of our center in the China Liver Transplant Registry database.

### Surgery and postoperative management

All patients underwent deceased donor LT using standard techniques without the use of veno-venous by-pass, and the piggyback technique was used when necessary. The post-LT immune suppression regimen included steroids and tacrolimus or cyclosporine with or without mycophenolate. Steroids were usually discontinued within the first month after operation. Postoperative adjuvant chemotherapy was administered according to tumor stage, physical condition, and the patient’s willingness. An anesthesiologist and circuit nurse added the blood in laparotomy sponges to suction containers and IBL was calculated immediately after surgery. A transfusion event was considered as administration of allogeneic packed red blood cells.

### Follow-up

The follow-up started on the day of LT and was routinely performed at outpatient clinics. Patients were followed up every 2 months for the first year after LT, and then at least every 3–4 months thereafter until death or the end of the study (December 31, 2014). Serum α-fetoprotein (AFP) levels, abdominal ultrasonography, and chest X-ray were monitored at each visit. When tumor recurrence was suspected, computed tomography and/or magnetic resonance imaging were performed to verify whether intrahepatic recurrence and/or distal metastasis had occurred. A diagnosis of recurrence was based on typical imaging appearance on computed tomography and/or magnetic resonance imaging scans, with or without raised AFP levels.

### Statistical analysis

Categorical variables are reported as frequencies (%) and were analyzed by the chi-square test. Continuous variables are shown as mean ± SD and were analyzed using the Student’s *t*-test. Patients without evidence of HCC recurrence at the last follow-up visit were right-censored. The recurrence-free survival rate and recurrence rate were estimated using the Kaplan–Meier method, and differences in the cumulative recurrence curves were compared by the log-rank test. The Cox proportional hazards regression model was used to identify independent prognostic factors for recurrence. In the analysis for early (within 1 year) HCC recurrence, follow-up time of the first postoperative year was used and patients who developed HCC recurrence after the first year were right-censored. Interaction between IBL and tumor characteristics was investigated using the Cox proportional hazard regression model. Independent risk factors for IBL > 4 L were determined by multivariate logistic regression analysis. All of the data were analyzed using the software package SPSS (version 22.0; SPSS Inc., Chicago, IL, USA) and the level of significance was set at *P* < 0.05.

## Results

### Patients’ characteristics

The patients’ characteristics are shown in Table 1. The mean age of the 479 patients was 55.7 years (range: 25–84 years) and there was a male predominance (*n* = 435, 90.8 %). The most common cause for LT was hepatitis B (*n* = 446, 93.1 %). Pre-transplant tumor therapy for HCC was performed in 176 (36.7 %) patients. Of these patients, 54 (11.3 %) had liver resection, 116 (24.2 %) had transhepatic arterial chemotherapy and embolization, 23 (4.8 %) had radiofrequency ablation, and 12 (2.5 %) had percutaneous ethanol injection before transplantation. Liver resection and transhepatic arterial chemotherapy and embolization were the main downstaging procedures for patients exceeding the Milan criteria. Among 126 patients receiving downstaging procedures, 41 (32.5 %) met the Milan criteria after treatments. Fourteen (2.9 %) patients underwent LT using the piggyback technique. The mean IBL was 1436 ± 1833 mL, and 359 (74.9 %) patients received perioperative allogeneic red blood cell transfusion. Of the 479 patients, 292 patients (61.0 %) were beyond the Milan criteria.

**Table 1:** Clinicopathological variables

Variable	Value
Age (years)	55.7 ± 8.8
Male sex	435 (90.8)
Hepatitis B virus infection	446 (93.1)
Preoperative tumor therapy	176 (36.7)
Child–Pugh score	7.7 ± 2.3
MELD score	12.8 ± 5.8
Piggyback LT	14 (2.9)
Number of tumors > 3	135 (28.2)
Maximum tumor size (cm)	4.8 ± 4.1
AFP levels > 400 ng/mL	176 (36.7)
Poor differentiation	60 (12.5)
Macrovascular invasion	69 (14.4)
Microvascular invasion	134 (28.0)
Beyond Milan criteria	292 (61.0)
Operation time (h)	8.0 ± 1.6
Blood transfusion(yes vs no)	359 (74.9)
Blood transfusion (mL)	1436.8 ± 1833.8
IBL (mL)	2172.5 ± 1854.0

Values are mean ± SD or n (%)

**Fig. 1:**
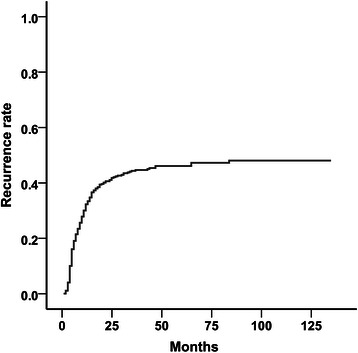
Kaplan–Meier curves for cumulative recurrence rates after liver transplantation for the entire patient cohort. The 1-, 3-, and 5-year recurrence-free survival rates were 33.3 %, 44.4 %, and 46.2 %, respectively

The median follow-up time for all of the patients who were enrolled in this study was 28 months (range, 1–131 months). The 1-, 3-, and 5-year recurrence rates were 33.3, 44.4, and 46.2 %, respectively (Fig. 1). During the follow-up period, 207 (43.2 %) patients experienced recurrence. Most HCC recurrences (*n* = 189, 91.3 %) occurred within 2 years, and 154 (74.4 %) patients experienced HCC recurrence within 1 year. The median time to recurrence was 7 months (range, 1–83 months).

### Relation between IBL and HCC recurrence after LT

**Fig. 2:**
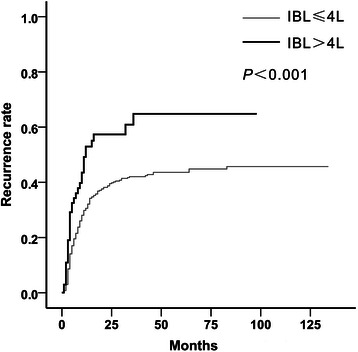
Kaplan–Meier curves for cumulative recurrence rates according to IBL. One- and 3-year recurrence rates were 30.5 % and 42.0 % for IBL ≤ 4 L, and 52.6 and 62.8 % for IBL > 4 L, respectively

Patients were categorized according to IBL at per liter intervals. Kaplan–Meier analysis with the log-rank test was used. One- and 3-year recurrence rates were 30.9 and 44.7 % for IBL < 1 L, 28.1 and 37.3 % for IBL of 1–2 L, 37.5 and 48.4 % for IBL of 2–3 L, 33.1 and 43.0 % for IBL of 3–4 L, and 52.6 and 62.8 % for IBL > 4 L. IBL > 4 L was significantly associated with a poorer recurrence-free survival (*P* < 0.001 for IBL > 4 L vs. ≤ 4 L, Fig. 2). Based on these results, the threshold for IBL was set at 4 L. Of the 479 patents, 69 (14.4 %) had an IBL > 4 L. The model for end-stage liver disease (MELD) score (*P* < 0.001), the presence of ascites (*P* < 0.001), blood transfusion (*P* < 0.001), and operation time (*P* = 0.029) were significantly different between patients with IBL > 4 L and those with IBL ≤ 4 L (Table 2). Patients’ characteristics, such as age, sex, and preoperative tumor therapy, tumor factors, such as the number of tumors, maximum tumor size, vascular invasion, and tumor differentiation, and serum AFP levels were not significantly different between the two groups. In univariate analysis, a total of nine factors, including IBL, had a significant effect on HCC recurrence (Table 3). In multivariate analysis, IBL > 4 L (*P* < 0.001; hazard ratio [HR] = 2.32; 95 % confidence interval [CI] = 1.60–3.36) was still a significant prognostic factor of recurrence for HC, as well as age < 60 years (*P* = 0.033; HR = 1.43; 95 % CI = 1.03–1.99), exceeding the Milan criteria (*P* < 0.001; HR = 2.54; 95 % CI = 1.79–3.59), AFP levels > 400 ng/mL (*P* < 0.001; HR = 1.65; 95 % CI = 1.24–2.19), and microvascular (*P* < 0.001; HR = 1.84; 95 % CI = 1.38–2.47) and macrovascular (*P* < 0.001; HR = 3.19; 95 % CI = 2.30–4.12) invasion (Table 3).

**Table 2:** Clinicopathological characteristics according to IBL

Variable	IBL ≤ 4 L	IBL > 4 L	*P* value
	(*n* = 410)	(*n* = 69)	
Mean age (years)	55.8 ± 9.1	56.5 ± 8.1	0.545
Male sex	372 (90.7)	63 (91.3)	0.879
Hepatitis B virus infection	383 (93.4)	63 (91.3)	0.522
Piggyback LT	12(2.9)	2(2.9)	0.990
Child-Pugh score	7.36 ± 2.13	9.21 ± 2.38	0.245
MELD score	12.3 ± 5.6	17.8 ± 8.8	<0.001
Ascites	205 (50.0)	55 (79.7)	<0.001
Variceal bleeding	49 (12.0)	15 (21.7)	0.027
Preoperative tumor therapy	146 (35.6)	29 (42.0)	0.498
History of abdominal surgery	85 (20.7)	20 (29.0)	0.125
AFP levels > 400 ng/mL	151 (36.8)	25 (36.2)	0.924
Size of largest tumor > 5 cm	122 (29.8)	17 (24.6)	0.386
Number of tumors > 3	117 (28.5)	18 (26.1)	0.676
Macrovascular invasion	55 (13.4)	14 (20.3)	0.103
Microvascular invasion	113 (27.6)	21 (30.4)	0.623
Poor differentiation	50 (12.2)	10 (14.5)	0.594
Beyond Milan criteria	248 (60.4)	44 (63.7)	0.567
Blood transfusion (mL)	1043.6 ± 1467.9	3892.2 ± 1880.8	<0.001
Operation time (h)	7.8 ± 1.5	8.9 ± 1.9	0.029

Values are mean ± SD or n (%)

**Table 3:** Predictors of recurrence after LT for HCC

Variable	Univariate analysis	Multivariate analysis
	*P*	HR (95 % CI) *P*
Age <60 years	0.004	1.43 (1.03–1.99) 0.033
Male sex	0.025	1.07 (0.62–1.86) 0.808
Hepatitis B virus infection	0.604	
Pre-LT tumor therapy	0.126	
MELD score > 15	0.472	
AFP levels > 400 ng/mL	<0.001	1.65 (1.24–2.19) 0.001
Poor differentiation	0.030	1.26 (0.84–1.88) 0.263
Macrovascular invasion	<0.001	3.19 (2.30–4.12) <0.001
Microvascular invasion	<0.001	1.84 (1.38–2.47) <0.001
Beyond Milan criteria	<0.001	2.54 (1.79–3.59) <0.001
Blood transfusion	0.496	
IBL > 4 L	<0.001	2.32 (1.60–3.36) <0.001
Operation time > 8 h	0.128	

### Relation between IBL and early recurrence of HCC

Approximately three-quarters of recurrence (*n* = 154, 74.4 %) occurred within 1 year. Therefore, we also evaluated risk factors contributing to early (within 1 year) recurrence. Cox regression analysis was performed considering patients without tumor recurrence within 1 year as right-censored. In multivariate analysis, IBL > 4 L was an independent predictor of early recurrence (*P* < 0.001; HR = 2.45; 95 % CI = 1.64–3.66), as well as the Milan criteria (*P* < 0.001; HR = 2.12; 95 % CI = 1.41–3.20), AFP levels > 400 ng/mL (*P* = 0.001; HR = 1.71; 95 % CI = 1.24–2.78), and microvascular (*P* < 0.001; HR = 1.94; 95 % CI = 1.40–2.71) and macrovascular (*P* < 0.001; HR = 3.26; 95 % CI = 2.29–4.64) invasion (Table 4).

**Table 4:** Predictors of early recurrence after LT for HCC

Variable	Univariate analysis	Multivariate analysis
	*P*	HR (95 % CI) *P*
Age <60 years	0.022	1.27 (0.87–1.86) 0.217
Male sex	0.080	
Hepatitis B virus infection	0.712	
Pre-LT tumor therapy	0.203	
AFP levels > 400 ng/mL	<0.001	1.71 (1.24–2.78) 0.001
Poor differentiation	0.020	1.31 (0.84–2.03) 0.232
Macrovascular invasion	<0.001	3.26 (2.29–4.64) <0.001
Microvascular invasion	<0.001	1.94 (1.40–2.71) <0.001
Beyond Milan criteria	<0.001	2.12 (1.41–3.20) <0.001
Blood transfusion	0.077	
IBL > 4 L	<0.001	2.45 (1.64–3.66) <0.001

### Stratified analysis of IBL according to tumor characteristics

**Fig. 3:**
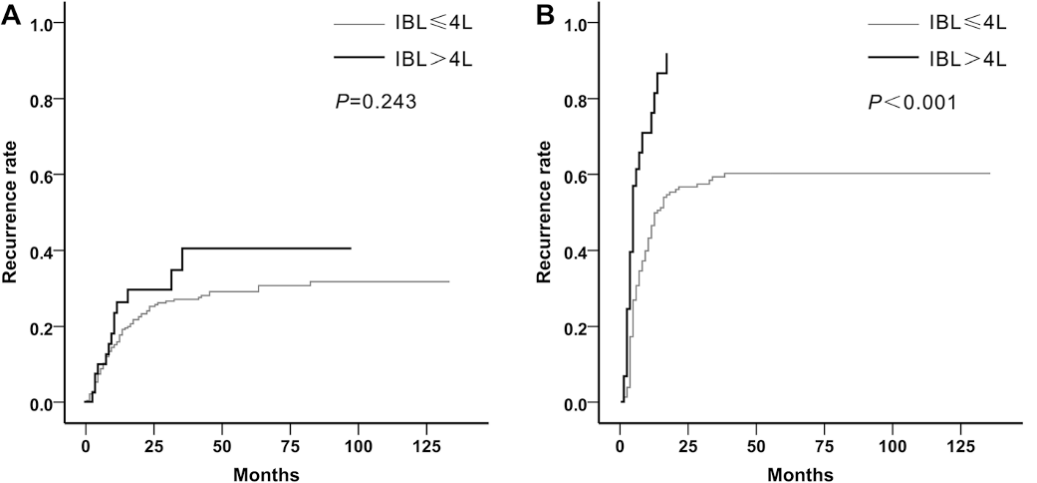
Kaplan–Meier curves for cumulative recurrence rates classified according to IBL and vascular invasion status. IBL > 4 L was not significantly associated with HCC recurrence in patients without vascular invasion (**a**). However, in patients with vascular invasion, IBL > 4 L predicted a significantly higher recurrence rate (**b**)

For patients within the Milan criteria, the 1-, 3-, and 5-year recurrence rates were 13.5, 24.2 %, and 27.8 %, respectively. For patients beyond the Milan criteria, the 1-, 3-, and 5-year recurrence rates were 53.5, 56.0, and 66.7 %, respectively. To further examine the relationship between IBL and recurrence-free survival, we performed stratified analysis according to the Milan criteria, status of vascular invasion, AFP levels, and tumor differentiation. When patients were categorized according to the vascular invasion status, IBL > 4 L (*P* = 0.243) was no longer associated with a high recurrence rate in patients without vascular invasion. However, for patients with vascular invasion, IBL > 4 L (*P* < 0.001) was still significantly associated with a higher recurrence rate (Fig. 3). Furthermore, stratified analysis suggested a significant interaction between IBL > 4 L and vascular invasion (*P* = 0.019, Table 5). In multivariate analysis for patients with vascular invasion, IBL > 4 L (*P* < 0.001; HR = 2.86, 95 % CI = 1.76–4.64) was an independent predictor of recurrence. However, for patients without vascular invasion, IBL > 4 L (*P* = 0.138; HR = 1.57, 95 % CI = 0.87–2.85) was not associated with HCC recurrence (Table 6).

**Table 5:** Stratified analysis for IBL > 4 L according to tumor characteristics

	HR	95 % CI	*P* value for interaction
AFP levels			0.671
≤ 400 ng/mL	1.88	1.14–3.10	
> 400 ng/mL	2.05	1.19–3.53	
Differentiation			0.309
Well/moderate	1.79	1.21–2.66	
Poor	2.58	0.96–6.92	
Vascular invasion			0.019
No	1.42	0.79–2.55	
Yes	2.91	1.80–4.69	
Milan criteria			0.565
Within	1.84	1.19–2.83	
Beyond	2.43	1.20–4.93

**Table 6:** Predictors of recurrence after LT for HCC according to vascular invasion

	Vascular invasion (*n* = 172)			Non-vascular invasion (*n* = 307)		
	HR	95 % CI	*P* value	HR	95 % CI	*P* value
Age <60 years	1.46	0.91–2.34	0.116	1.40	0.87-2.25	0.167
AFP levels > 400 ng/mL	1.53	1.04–2.24	0.031	1.99	1.31–3.02	0.001
Poor differentiation	1.09	0.68–1.73	0.729	1.37	0.63–2.98	0.423
Beyond Milan criteria	3.01	1.94–4.87	<0.001	1.38	0.90–2.10	0.139
IBL > 4 L	2.86	1.76–4.64	<0.001	1.57	0.87–2.85	0.138

### Risk factors for excessive IBL

Our results showed that excessive IBL, particularly IBL > 4 L, was a significant predictor for a higher recurrence rate and early recurrence. Therefore, we evaluated the potential risk factors related to IBL > 4 L. Clinicopathological characteristics of patients with IBL ≤ 4 L were compared with those with IBL > 4 L previously in our study (Table 2). There were significant differences in the history of variceal bleeding, the presence of ascites, and the MELD score between the two groups. In multivariate analysis using logistic regression analysis, the presence of ascites (*P* = 0.006; HR = 2.61; 95 % CI = 1.32–5.14) and a MELD score > 15 (*P* < 0.001; HR = 3.25; 95 % CI = 1.84–5.75) were independent risk factors for IBL > 4 L (Table 7).

**Table 7:** Multivariate analysis of risk factors for IBL > 4 L

Variable	HR	95 % CI	*P* value
History of variceal bleeding	1.51	0.75–3.07	0.253
History of abdominal surgery	1.68	0.89–3.15	0.108
Ascites	2.61	1.32–5.14	0.006
MELD score > 15	3.25	1.84–5.75	<0.001

## Discussion

Characteristics of tumors have been shown to be of prognostic importance for HCC patients after LT [8–11]. However, other factors may also affect HCC recurrence. In this study, we assessed the relationship between IBL and HCC recurrence after LT through comprehensive evaluation. We found that excessive blood loss, particularly IBL > 4 L, was an independent predictor of HCC recurrence, especially in patients with vascular invasion.

Previous studies have evaluated the relationship of IBL with outcome in HCC patients after hepatectomy. Katzet et al. [3] reported that an IBL volume greater than 1 L during liver resection was significantly associated with poor overall survival, disease-specific survival, and recurrence-free survival. Taketomi et al. [12] found that IBL > 1 L was a significant predictor of extrahepatic recurrence following hepatectomy. Sasaki et al. [13] demonstrated that IBL > 1 L was a predictive factor of cancer-related death within 2 years after hepatectomy for HCC patients without microvascular invasion. However, all of these studies focused on the prognostic value of IBL after liver resection rather than liver transplantation. Previous studies have identified that IBL during LT has adverse effects on post-transplant outcome [14–17]. However, the results from these studies were mainly obtained from data of patients with benign end-stage liver diseases, without considering HCC patients independently, as well as the relationship between IBL and tumor recurrence. Recently, a study from our center demonstrated that IBL during LT was a significant prognostic factor for long-term survival of HCC patients after LT [18]. Therefore, we further assessed the prognostic value of IBL on HCC recurrence after LT. By using threshold analysis, we found that when IBL was > 4 L, patients showed significantly higher recurrence rates. In multivariate analysis, IBL > 4 L was a significant prognostic factor for cumulative recurrence rates and early recurrence. We also found that tumor characteristics, including serum AFP levels, either macro- or microvascular invasion, and the Milan criteria, were significant independent predictors of HCC recurrence after LT, which is consistent with previous reports [11, 19, 20]. Additionally, we found that patients aged younger than 60 years had higher recurrence rates, probably because of more advanced and aggressive HCC in young people [21].

Previous studies have suggested that perioperative blood transfusion is significantly associated with HCC recurrence after LT [17, 22, 23]. However, we did not find any adverse effects of packed red blood cell transfusion on outcome in our study. In contrast, excessive IBL was a significant predictor of outcome by univariate and multivariate analyses. Because not all of these previous studies included IBL in their analysis, we speculate that the correlation between transfusion and poor oncological outcome might be partly because of unmeasured effects of IBL [3]. IBL is probably a more sensitive predictor of HCC recurrence than blood transfusion.

Because excessive IBL was an important prognostic factor of HCC recurrence after LT, the potential factors affecting profuse hemorrhage need to be investigated. Although blood loss during LT is multifactorial, the major causes of excessive IBL are technical difficulties and pre-existing abnormalities of the hemostatic system [24]. Previous studies have shown that the presence of ascites [25], preoperative MELD score [26], and operation time [16] are risk factors of IBL during LT. Similar to these studies, these factors were determined as independent risk factors of excessive IBL in our study. The presence of ascites and a high MELD score preoperatively indicate more severe liver dysfunction. Additionally, there might be a relationship between ascites and fibrinolytic activity [27]; therefore, severe coagulopathy could be aggravated by ascites.

Although we showed that excessive blood loss was an independent predictor of HCC recurrence after LT, we cannot conclude that excessive IBL directly leads to a worse cancer outcome. There are several potential explanations. Excessive IBL is a consequence of mixed factors and reflects worse pre-transplant conditions combined with technical difficulties. This condition also reflects more manipulation of the liver at the time of hepatectomy, which possibly leads to more tumor shedding at that time [3]. However, profuse hemorrhage may result in tissue hypoperfusion and hypoxia for a long time, which in turn may lead to systemic inflammation and impaired antitumor immunity [28]. Additionally, a hypoxic environment could promote epithelial-mesenchymal transition, which makes the residual tumor cells more aggressive. Because patients with vascular invasion are more likely to harbor micrometastases, the effect of excessive IBL should be greater in these patients. Our study verified this hypothesis. By performing stratified analysis, we found a significant interaction between excessive IBL and vascular invasion. For patients with vascular invasion, IBL > 4 L was an independent predictor of HCC recurrence, in addition to elevated AFP levels and exceeding the Milan criteria.

Several limitations of our study should be noted. First, as a retrospective study, there was a potential for inclusion, exclusion, and recall bias of patients and data. Second, considering that IBL was estimated by the anesthesiologist and circuit nurse, the actual degree of IBL may have been underestimated or overestimated. Assessment of IBL using the concept of red cell mass, which was calculated based on the perioperative change in hematocrit, might be more precise [29]. However, this method is impractical because assessment of hematocrit is not routinely conducted in our center. Additionally, because of the small number of patients (*n* = 69, 14.4 %) in our single center, further multicenter studies are required to confirm the relationship between IBL and post-transplant tumor recurrence.

## Conclusions

This study shows that excessive IBL, particularly IBL > 4 L, is an independent predictor of HCC recurrence after LT. Excessive blood loss results in a significantly worse outcome, especially in patients with vascular invasion. These findings highlight the importance of minimizing operative blood loss and imply that patients with excessive blood loss should be monitored more closely after LT.

## Abbreviations

IBL: Intraoperative blood loss
HCC: Hepatocellular carcinoma
LT: Liver transplantation
MELD: Model for end-stage liver disease
AFP: α-Fetoprotein

## References

[1] Bruix J, Gores GJ, Mazzaferro V (2014). Hepatocellular carcinoma: clinical frontiers and perspectives. Gut.

[2] Oliveri RS, Wetterslev J, Gluud C (2012). Hepatocellular carcinoma. Lancet.

[3] Katz SC, Shia J, Liau KH, Gonen M, Ruo L, Jarnagin WR (2009). Operative blood loss independently predicts recurrence and survival after resection of hepatocellular carcinoma. Ann Surg.

[4] Liang YX, Guo HH, Deng JY, Wang BG, Ding XW, Wang XN (2013). Impact of intraoperative blood loss on survival after curative resection for gastric cancer. World J Gastroenterol.

[5] Morner ME, Gunnarsson U, Jestin P, Svanfeldt M (2012). The importance of blood loss during colon cancer surgery for long-term survival: an epidemiological study based on a population based register. Ann Surg.

[6] Oefelein MG, Colangelo LA, Rademaker AW, McVary KT (1995). Intraoperative blood loss and prognosis in prostate cancer patients undergoing radical retropubic prostatectomy. J Urol.

[7] Nagai S, Fujii T, Kodera Y, Kanda M, Sahin TT, Kanzaki A (2011). Impact of operative blood loss on survival in invasive ductal adenocarcinoma of the pancreas. Pancreas.

[8] Mazzaferro V, Regalia E, Doci R, Andreola S, Pulvirenti A, Bozzetti F (1996). Liver transplantation for the treatment of small hepatocellular carcinomas in patients with cirrhosis. N Engl J Med.

[9] Yao FY, Ferrell L, Bass NM, Watson JJ, Bacchetti P, Venook A (2001). Liver transplantation for hepatocellular carcinoma: expansion of the tumor size limits does not adversely impact survival. Hepatology.

[10] Zheng SS, Xu X, Wu J, Chen J, Wang WL, Zhang M (2008). Liver transplantation for hepatocellular carcinoma: Hangzhou experiences. Transplantation.

[11] Mazzaferro V, Llovet JM, Miceli R, Bhoori S, Schiavo M, Mariani L (2009). Predicting survival after liver transplantation in patients with hepatocellular carcinoma beyond the Milan criteria: a retrospective, exploratory analysis. Lancet Oncol.

[12] Taketomi A, Toshima T, Kitagawa D, Motomura T, Takeishi K, Mano Y (2010). Predictors of extrahepatic recurrence after curative hepatectomy for hepatocellular carcinoma. Ann Surg Oncol.

[13] Sasaki K, Matsuda M, Ohkura Y, Kawamura Y, Inoue M, Hashimoto M (2014). Factors associated with early cancer-related death after curative hepatectomy for solitary small hepatocellular carcinoma without macroscopic vascular invasion. J Hepatobiliary Pancreat Sci.

[14] Shaw BW, Wood RP, Gordon RD, Iwatsuki S, Gillquist WP, Starzl TE (1985). Influence of selected patient variables and operative blood loss on six-month survival following liver transplantation. Semin Liver Dis.

[15] Mor E, Jennings L, Gonwa TA, Holman MJ, Gibbs J, Solomon H (1993). The impact of operative bleeding on outcome in transplantation of the liver. Surg Gynecol Obstet.

[16] Rana A, Petrowsky H, Hong JC, Agopian VG, Kaldas FM, Farmer D (2013). Blood transfusion requirement during liver transplantation is an important risk factor for mortality. J Am Coll Surg.

[17] Cywinski JB, Alster JM, Miller C, Vogt DP, Parker BM (2014). Prediction of intraoperative transfusion requirements during orthotopic liver transplantation and the influence on postoperative patient survival. Anesth Analg.

[18] Teng F, Wang GH, Tao YF, Guo WY, Wang ZX, Ding GS (2014). Criteria-specific long-term survival prediction model for hepatocellular carcinoma patients after liver transplantation. World J Gastroenterol.

[19] Lim KC, Chow PK, Allen JC, Chia GS, Lim M, Cheow PC (2011). Microvascular invasion is a better predictor of tumor recurrence and overall survival following surgical resection for hepatocellular carcinoma compared to the Milan criteria. Ann Surg.

[20] Duvoux C, Roudot-Thoraval F, Decaens T, Pessione F, Badran H, Piardi T (2012). Liver transplantation for hepatocellular carcinoma: a model including alpha-fetoprotein improves the performance of Milan criteria. Gastroenterology.

[21] Huang J, Li BK, Chen GH, Li JQ, Zhang YQ, Li GH (2009). Long-term outcomes and prognostic factors of elderly patients with hepatocellular carcinoma undergoing hepatectomy. J Gastrointest Surg.

[22] Kaido T, Takada Y, Egawa H, Uemoto S (2009). The influence of intraoperative homologous blood transfusion on prognosis after liver transplantation for hepatocellular carcinoma. Hepatogastroenterology.

[23] Nagai S, Yoshida A, Facciuto M, Moonka D, Abouljoud MS, Schwartz ME (2015). Ischemia time impacts recurrence of hepatocellular carcinoma following liver transplantation. Hepatology.

[24] Feltracco P, Brezzi M, Barbieri S, Galligioni H, Milevoj M, Carollo C (2013). Blood loss, predictors of bleeding, transfusion practice and strategies of blood cell salvaging during liver transplantation. World J Hepatol.

[25] Xia VW, Fond A, Du B (2006). Ascites, but Not Hyponatremia, Is Associated With High Intraoperative Transfusion and Vasopressor Requirements During Liver Transplantation. Transplant Proc.

[26] Yuasa T, Niwa N, Kimura S, Tsuji H, Yurugi K, Egawa H (2005). Intraoperative blood loss during living donor liver transplantation: an analysis of 635 recipients at a single center. Transfusion.

[27] Agarwal S, Joyner KA, Swaim MW (2000). Ascites fluid as a possible origin for hyperfibrinolysis in advanced liver disease. Am J Gastroenterol.

[28] Jubert AV, Lee ET, Hersh EM, McBride CM (1973). Effects of surgery, anesthesia and intraoperative blood loss on immunocompetence. J Surg Res.

[29] Bang SR, Ahn HJ, Kim GS, Yang M, Gwak MS, Ko JS (2010). Predictors of high intraoperative blood loss derived by simple and objective method in adult living donor liver transplantation. Transplant Proc.

